# Association between total sugar intake and all-cause mortality according to bone health status: A retrospective cohort study

**DOI:** 10.1097/MD.0000000000046133

**Published:** 2026-01-23

**Authors:** Tingting Chen, Min Liang

**Affiliations:** a Department of Geriatric Endocrinology and Metabolism, The First Affiliated Hospital of Guangxi Medical University, Nanning, Guangxi, China.

**Keywords:** mortality, NHANES, osteopenia, osteoporosis, sugar intake

## Abstract

The relationship between total sugar intake and all-cause mortality remains insufficiently understood. This study aimed to evaluate the association between total sugar intake and all-cause mortality among individuals with varying bone health status. This retrospective cohort study was based on data from the National Health and Nutrition Examination Survey. A total of 15,886 participants were included, comprising 9783 individuals with normal bone density and 6103 with osteopenia or osteoporosis. Cox proportional hazards regression models and Kaplan–Meier curves were used to assess the association between total sugar intake and all-cause mortality across different bone health statuses. Restricted cubic splines were applied to explore potential nonlinear relationships. Subgroup analyses were performed based on age, sex, body mass index, diabetes, and hypertension. Significant differences in demographics, lifestyle habits, medical history, and mortality were observed between individuals with normal bone density and those with osteopenia or osteoporosis. After adjusting for potential confounders, the hazard ratios for all-cause mortality across tertiles of total sugar intake were 1.000, 1.067 (95% confidence interval: 0.890–1.279), and 1.279 (95% confidence interval: 1.076–1.522), respectively (*P* for trend = .013). Additionally, each 10-unit increase in total sugar intake was associated with a 2.9% increase in all-cause mortality risk (*P* < .001). Notably, this association was significant only among participants with osteopenia or osteoporosis, but not among those with normal bone density. The results were consistent across most subgroups. Higher total sugar intake was associated with increased all-cause mortality among individuals with compromised bone health (osteopenia or osteoporosis), but not among those with normal bone density.

## 1. Introduction

Osteoporosis is a prevalent systemic skeletal disorder characterized by reduced bone mass and deterioration of the bone microarchitecture, leading to increased susceptibility to fragility fractures and elevated mortality risk.^[1]^ With global population aging, the burden of osteoporosis and related fractures continues to rise. In 2010, osteoporosis and low bone mass affected approximately 53.6 million U.S. adults aged 50 and older. This number is projected to rise by 17.2 million from 2010 to 2030.^[2]^ Early identification and intervention are critical for preventing fractures, and dietary modification is widely recognized as a practical and effective strategy for promoting bone health.^[3]^

Sugar, while a common component of the human diet, has become a growing public health concern due to its overconsumption worldwide. Excessive intake of dietary sugar has been strongly associated with numerous adverse health outcomes, including obesity, type 2 diabetes, cardiovascular disease (CVD), hyperuricemia, dental caries, and certain cancers.^[4]^ In response, the World Health Organization (WHO) recommends that added sugars should contribute <10% of total daily energy intake for both adults and children.^[5,6]^

Although excessive sugar intake and the rising prevalence of osteoporosis are both major public health challenges, the relationship between sugar consumption and bone health remains insufficiently understood. In particular, it is unclear whether sugar intake has differential effects on mortality risk depending on bone health status. Therefore, the present study aimed to examine the association between total sugar intake and all-cause mortality, stratified by bone health status.

## 2. Methods

### 2.1. Study design and population

**Figure 1.**
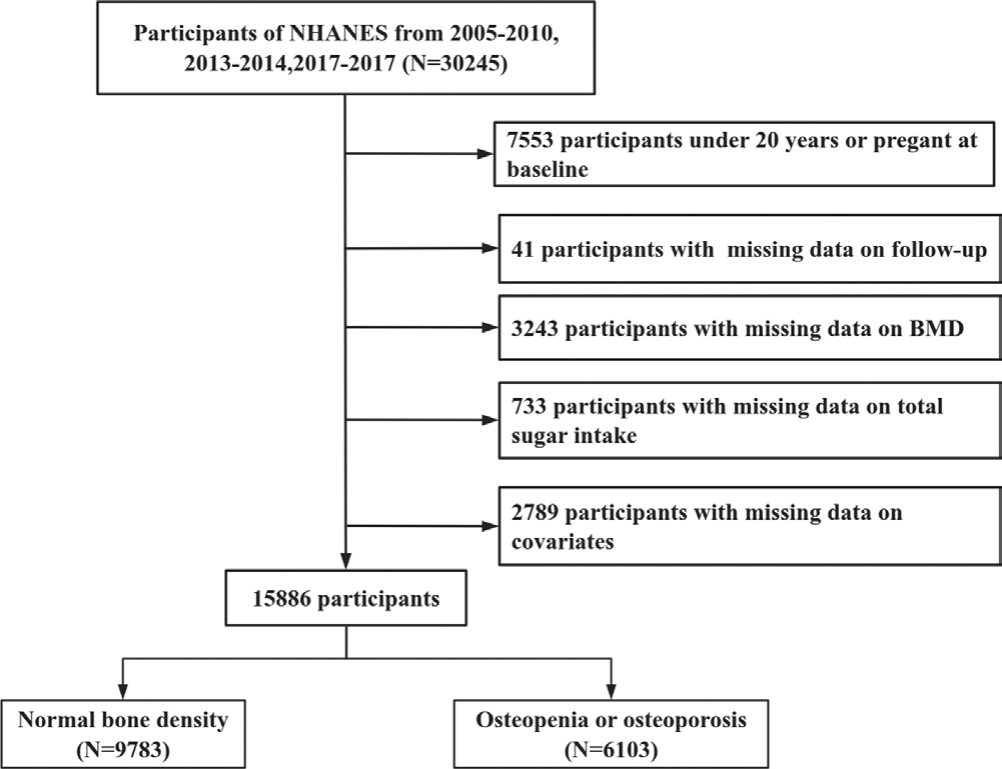
Flowchart of participants selection.

This study was based on data from the National Health and Nutrition Examination Survey (NHANES), which is publicly available and can be freely accessed and downloaded from the official website (https://wwwn.cdc.gov/nchs/nhanes/Default.aspx). Written informed consent was obtained from all participants at the time of data collection, and the NHANES study protocol was approved by the Research Ethics Review Board of the National Center for Health Statistics. For the present analysis, we included included 15,886 individuals from the 2005 to 2010, 2013 to 2014, and 2017 to 2018 NHANES cycles. Eligible participants were aged 20 years or older, nonpregnant, and had complete data on bone mineral density (BMD), total sugar intake, relevant covariates, and survival status. The participant selection process is illustrated in Figure 1.

### 2.2. Outcome

The primary outcome was all-cause mortality, determined by linkage to the National Death Index through December 31, 2019. Causes of death were classified according to the International Classification of Diseases, 10th Revision, and included malignant neoplasms, CVD, respiratory diseases, Alzheimer disease, diabetes, nephropathy-related conditions, unintentional injuries, and other causes.

### 2.3. Bone health status assessment

BMD measurements were used to assess bone health status. *T*-scores were calculated for each participant at each skeletal site using the formula: (BMDpatient − µreference)/σreference, where µreference and σreference represent the mean and standard deviation of BMD values from the reference population. In accordance with the WHO guidelines, the reference group for total hip and femoral neck *T*-score calculations comprised non-Hispanic white women aged 20 to 29 years, as reported in NHANES III.^[7,8]^ Bone health status was classified based on the WHO criteria: normal bone density (*T*-score ≥ −1.0), osteopenia (−2.5 < *T*-score < −1.0), and osteoporosis (*T*-score ≤ −2.5).^[9]^

### 2.4. Total sugar intake assessment

Dietary intake was assessed using two 24-hour dietary recalls for each participant. The first recall was conducted in person at the Mobile Examination Center (MEC) by trained interviewers, during which participants reported all foods, beverages, and dietary supplements consumed from midnight to midnight of the preceding day. The second recall was conducted by telephone 3 to 10 days later. Nutrient intake data, including total sugar, were derived using the United States Department of Agriculture Food and Nutrient Database for Dietary Studies.^[10]^ Total sugar intake was calculated as either the value from the first 24-hour recall alone or the average of both recalls, depending on data availability.

### 2.5. Covariates

Demographic variables included age (years), sex (male/female), race (Mexican American, Non-Hispanic Black, Non-Hispanic White, Other), and marital status (married, widowed, divorced, separated, never married, other). Socioeconomic status was represented by the family poverty-to-income ratio (≤1.3, 1.3–3.5, >3.5) and education level (elementary or less, high school or equivalent, college or above). Lifestyle-related variables included body mass index (BMI, kg/m²), physical activity (none, moderate, vigorous), smoking status (never, former, current), and alcohol consumption (yes/no).

Medical history variables included hypertension, diabetes mellitus, CVD, chronic kidney disease, dyslipidemia, cancer, history of fracture, and glucocorticoid use (all categorized as yes/no). Detailed definitions and measurement protocols for these variables are available on the NHANES website.

### 2.6. Statistics analysis

Baseline characteristics between the 2 groups were compared using the Wilcoxon rank-sum test for continuous variables and the Rao–Scott Chi-square test for categorical variables. Continuous variables were presented as median (25th–75th percentile), while categorical variables were expressed as unweighted counts with weighted percentages (n, [%]). To evaluate the association between total sugar intake and all-cause mortality, weighted Cox proportional hazards regression models were applied, and results were reported as hazard ratios (HRs) with 95% confidence intervals (CIs). Model 1 was unadjusted. Model 2 was adjusted for demographic and lifestyle factors, including age, sex, race, educational level, marital status, poverty-to-income ratio, smoking status, drinking status, and physical activity. Model 3 was further adjusted for clinical factors, including BMI, history of hypertension, CVD, diabetes mellitus, chronic kidney disease, dyslipidemia, cancer, fracture, and glucocorticoid use. Kaplan–Meier survival curves were plotted and stratified by tertiles of total sugar intake to visualize survival differences. Restricted cubic spline regression was employed to explore potential linear or nonlinear associations between total sugar intake and all-cause mortality, with statistical significance assessed using the Wald test. Additionally, subgroup analyses were conducted to assess effect modification across subpopulations stratified by age, sex, BMI, diabetes, and hypertension status. All statistical analyses were conducted using R software (version 4.3.2). A two-sided *P*-value < .05 was considered statistically significant.

## 3. Results

### 3.1. Baseline characteristics

The baseline characteristics of the participants are presented in Table 1. A total of 15,886 participants were included in this study, comprising 9783 individuals with normal bone density and 6103 individuals with osteopenia or osteoporosis. Participants with osteopenia or osteoporosis were generally older and had a higher proportion of females and Non-Hispanic Whites. Compared to those with normal bone density, this group exhibited lower BMI, lower rates of marriage and university-level education, and reduced physical activity. They also had a higher prevalence of poverty. Additionally, fewer participants with osteopenia or osteoporosis were current smokers or drinkers, but this group had a higher burden of comorbidities, including hypertension, CVD, chronic kidney disease, dyslipidemia, cancer, fractures, and prior glucocorticoid use. They also had a shorter follow-up duration and a higher all-cause mortality rate. The prevalence of diabetes mellitus did not differ significantly between the 2 groups.

**Table 1:** Basic characteristics and mortality for people grouped by bone health status.

Characteristics	Normal bone density	Osteopenia or osteoporosis	*P* value
N	9783	6103	
Age (yr)	47.00 (35.00, 58.00)	58.00 (48.00, 69.00)	<.001
BMI (kg/m^2^)	28.80 (25.30, 33.18)	26.06 (22.90, 29.82)	<.001
Female, n (%)	4113 (43.42)	3708 (63.91)	<.001
Marital status, n (%)			<.001
Married	5482 (59.38)	3291 (57.33)	
Widowed	505 (3.36)	894 (11.78)	
Divorced	1111 (10.89)	882 (14.70)	
Separated	330 (2.53)	174 (1.91)	
Never married	1572 (16.07)	577 (9.73)	
others	783 (7.77)	285 (4.55)	
Race, n (%)			<.001
Mexican American	1614 (7.75)	965 (6.30)	
Non-Hispanic White	4470 (70.19)	3375 (77.08)	
Non-Hispanic Black	2378 (12.54)	702 (5.43)	
Other Race	1321 (9.52)	1061 (11.19)	
Smoking status, n (%)			.006
Never	3015 (52.62)	2399 (51.88)	
Former	3038 (25.38)	2152 (28.52)	
Current	3730 (22.00)	1552 (19.59)	
Education level, n (%)			.014
Primary school and below	2344 (15.37)	1630 (16.62)	
High school or equivalent	2307 (23.74)	1483 (25.53)	
University education and above	5132 (60.89)	2990 (57.85)	
Poverty income ratio, n (%)			.001
≤1.3	2702 (18.51)	1787 (19.00)	
>1.3, ≤3.5	3675 (33.30)	2436 (37.10)	
>3.5	3406 (48.19)	1880 (43.90)	
Physical activity, n (%)			<.001
None	3015 (25.64)	2399 (32.18)	
Moderate	3038 (31.70)	2152 (38.15)	
Vigorous	3730 (42.66)	1552 (29.67)	
Drinking status, n (%)	7601 (81.58)	4279 (76.27)	<.001
History of hypertension, n (%)	4244 (39.26)	3141 (46.15)	<.001
History of cardiovascular disease, n (%)	981 (8.09)	924 (12.08)	<.001
History of chronic kidney disease, n (%)	1582 (12.03)	1395 (17.37)	<.001
History of dyslipidemia, n (%)	7357 (75.61)	4830 (79.42)	<.001
History of cancer, n (%)	869 (8.82)	886 (15.30)	<.001
History of fracture, n (%)	1012 (12.05)	924 (17.45)	<.001
History of diabetes mellitus, n (%)	1802 (13.95)	1096 (12.84)	.205
Use of glucocorticoid, n (%)	432 (4.68)	403 (7.36)	<.001
Total sugar intake (g)	101.42 (67.69, 148.36)	93.79 (63.73, 132.31)	<.001
All-cause mortality, n (%)	924 (6.93)	1135 (13.78)	<.001
Follow-up time (mo)	129.00 (80.00, 154.00)	115.00 (63.00, 144.00)	<.001

Continuous variables are expressed as the median (25th and 75th percentiles) and categorical variables are presented as unweighted counts with weighted percentages.

BMI = body mass index.

### 3.2. Associations between total sugar intake and all-cause mortality

**Figure 2.**
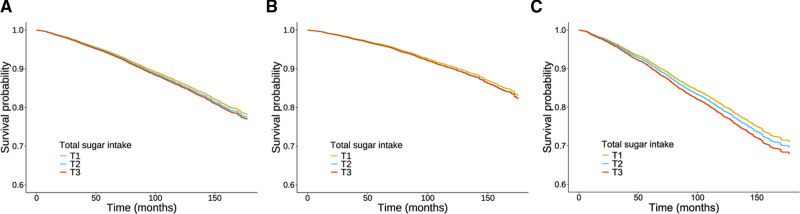
Kaplan–Meier curves of all-cause mortality in overall participants (A), participants with normal bone density (B), and participants with osteopenia or osteoporosis (C). Adjusted for age, gender, race, educational level, marital status, poverty income ratio, smoking statues, drinking status, physical activity, BMI, medical history of hypertension, cardiovascular disease, diabetes mellitus, chronic kidney disease, dyslipidemia, cancer, fracture, and use of glucocorticoid. BMI = body mass index.

The associations between total sugar intake and all-cause mortality are presented in Table 2. Among all participants, higher total sugar intake was associated with a lower risk of all-cause mortality in the unadjusted model (Model 1). However, this association became nonsignificant after adjustment for age, gender, race, educational level, marital status, poverty-income ratio, smoking status, alcohol consumption, and physical activity (Model 2). When further adjusted for BMI, medical history of hypertension, CVD, diabetes mellitus, chronic kidney disease, dyslipidemia, cancer, fractures, and glucocorticoid use (Model 3), the HRs for all-cause mortality across increasing tertiles of total sugar intake were 1.000 (reference), 1.156 (95% CI: 1.001–1.334), and 1.165 (95% CI: 1.020–1.331), respectively (*P* for trend = .030). Each 10-unit increase in total sugar intake was associated with a 1.2% increase in the risk of all-cause mortality (*P* = .003). In subgroup analyses by bone health status, participants with normal bone density initially showed an inverse association between total sugar intake and all-cause mortality in the unadjusted model (Model 1), but this association disappeared after adjusting for covariates in Models 2 and 3. In contrast, among participants with osteopenia or osteoporosis, no significant association was observed in the unadjusted model. However, in Model 2, the HRs for all-cause mortality across tertiles of total sugar intake were 1.000, 1.023 (95% CI: 0.861–1.216), and 1.206 (95% CI: 1.009–1.443), respectively (*P* for trend = .040). Each 10-unit increase in total sugar intake was associated with a 2.4% increase in mortality risk (*P* = .002). In Model 3, this association became stronger, with HRs of 1.000, 1.067 (95% CI: 0.890–1.279), and 1.279 (95% CI: 1.076–1.522), respectively (*P* for trend = .013). Each 10-unit increase in total sugar intake was associated with a 2.9% increase in all-cause mortality risk (*P* < .001). Kaplan–Meier survival curves stratified by total sugar intake tertiles are shown in Figure 2.

**Table 2:** Hazard ratios of mortality among participants by tertiles of total sugar intake.

All-cause mortality	Tertiles of total sugar intake			*P* for trend	Per 10 units increase	*P* value
	Tertile 1	Tertile 2	Tertile 3			
Overall						
Model 1	1.000	1.081 (0.904, 1.291)	0.760 (0.644, 0.897)	<.001	0.981 (0.971, 0.992)	<.001
Model 2	1.000	1.112 (0.968, 1.277)	1.081 (0.940, 1.243)	.234	1.009 (0.999, 1.018)	.068
Model 3	1.000	1.156 (1.001, 1.334)	1.165 (1.020, 1.331)	.030	1.012 (1.004, 1.021)	.003
Normal bone density						
Model 1	1.000	1.082 (0.828, 1.412)	0.677 (0.534, 0.859)	<.001	0.973 (0.958, 0.988)	<.001
Model 2	1.000	1.207 (0.954, 1.529)	0.978 (0.767, 1.247)	.997	0.996 (0.981, 1.011)	.584
Model 3	1.000	1.256 (0.979, 1.610)	1.084 (0.846, 1.390)	.367	1.001 (0.988, 1.015)	.871
Osteopenia or osteoporosis						
Model 1	1.000	1.128 (0.948, 1.344)	1.002 (0.819, 1.225)	.944	1.000 (0.986, 1.015)	.976
Model 2	1.000	1.023 (0.861, 1.216)	1.206 (1.009, 1.443)	.040	1.024 (1.009, 1.040)	.002
Model 3	1.000	1.067 (0.890, 1.279)	1.279 (1.076, 1.522)	.013	1.029 (1.015, 1.043)	<.001

Model 1 was unadjusted. Model 2 was adjusted for age, gender, race, educational level, marital status, poverty income ratio, smoking statues, drinking status and physical activity. Model 3 was further adjusted for BMI, medical history of hypertension, cardiovascular disease, diabetes mellitus, chronic kidney disease, dyslipidemia, cancer, fracture and use of glucocorticoid.

BMI = body mass index.

**Figure 3.**
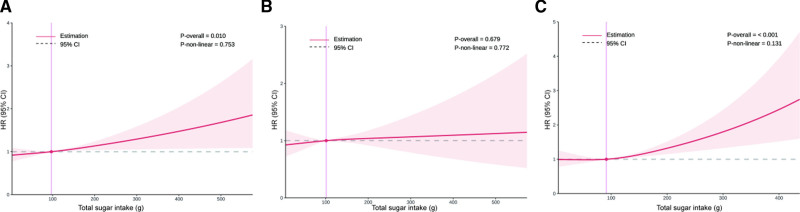
Hazard ratios for all-cause mortality by total sugar intake among overall participants (A), participants with normal bone density (B), and participants with osteopenia or osteoporosis (C). Adjusted for age, gender, race, educational level, marital status, poverty income ratio, smoking statues, drinking status, physical activity, BMI, medical history of hypertension, cardiovascular disease, diabetes mellitus, chronic kidney disease, dyslipidemia, cancer, fracture and use of glucocorticoid. BMI = body mass index.

Restricted cubic splines were used to explore the relationship between total sugar intake and all-cause mortality, as shown in Figure 3. Among all participants, the restricted cubic splines curves revealed a significant positive linear association between total sugar intake and all-cause mortality (*P* for overall = .001; *P* for nonlinearity = .753). A similar linear relationship was observed in participants with osteopenia or osteoporosis (*P* for overall < .001; *P* for nonlinearity = .131). In contrast, no significant or nonlinear association between total sugar intake and all-cause mortality among participants with normal bone density (*P* for overall = .679, *P* for nonlinear = .779).

### 3.3. Subgroup analyses

**Figure 4.**
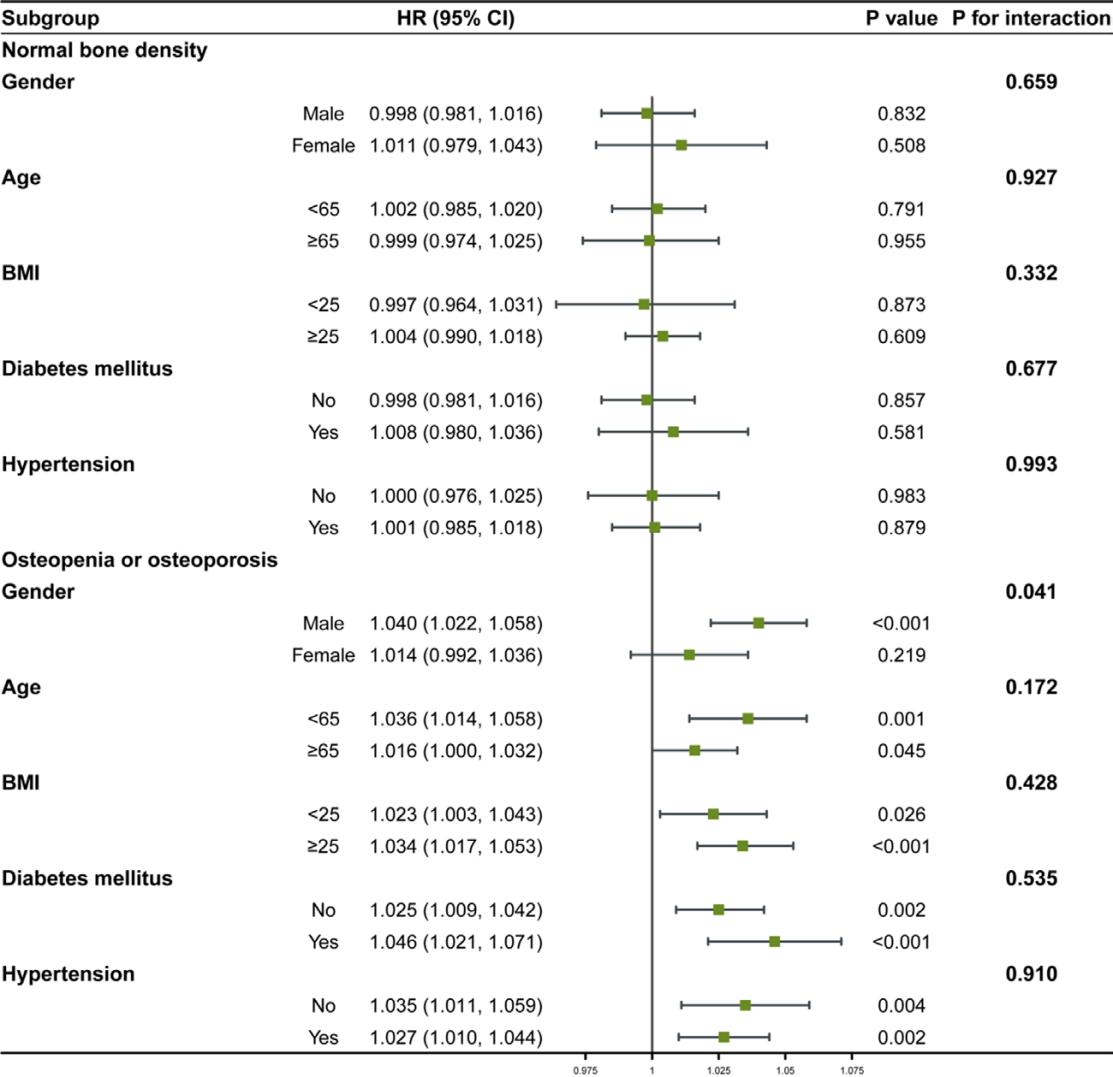
Subgroup analysis of total sugar intake of all-cause mortality.

Subgroup analyses were performed according to gender, age (<65, ≥65), BMI (<25, ≥25), and medical history of diabetes mellitus and hypertension. The results were presented in Tables S1 and S2, Supplemental Digital Content, https://links.lww.com/MD/R144 and illustrated as a forest plot in Figure 4. Among participants with osteopenia or osteoporosis, the positive association between total sugar intake and all-cause mortality risk remained consistent across most subgroups (all *P* for interaction > .05), with the exception of the female subgroup, where a significant interaction was observed (*P* for interaction = .041). In contrast, no significant associations were found in any of the subgroups among participants with normal bone density.

## 4. Discussion

This study investigated the association between total sugar intake and all-cause mortality, stratified by bone health status. We found that higher total sugar intake was significantly associated with an increased risk of all-cause mortality among individuals with osteopenia or osteoporosis, whereas no such association was observed in those with normal BMD after adjusting for potential confounders. These associations remained robust across most subgroups.

Excessive sugar consumption is widely recognized as a major contributor to various chronic diseases, including cardiometabolic conditions and malignancies, which are in turn linked to increased mortality risk.^[4]^ Our findings align with previous epidemiological studies reporting positive associations between sugar intake (particularly from sugar-sweetened beverages) and all-cause mortality in large cohorts such as the Dutch and UK Biobank populations.^[11–13]^ Similar trends were observed among cancer patients, where higher sugar intake was associated with greater cancer-specific and all-cause mortality.^[14,15]^ However, other studies have reported null associations, possibly due to low mortality rates or missing cardiovascular outcome data, which may have led to an underestimation of the true effect.^[16,17]^

A key novelty of our study lies in revealing that the effect of sugar intake on mortality risk is modified by bone health status. Osteoporosis is a common age-related metabolic bone disease that often coexists with other conditions such as CVD, diabetes, and dyslipidemia, all of which contribute to elevated mortality risk.^[18–20]^ Previous studies have reported inconsistent findings on the relationship between sugar intake and bone health. A meta-analysis of 6 studies found an inverse association between sugar-sweetened beverage consumption and BMD in adults.^[21]^ In contrast, a longitudinal study from China observed no significant link between sugar intake and bone loss or fracture risk in the elderly and even suggested a lower mortality rate with higher sugar intake.^[22]^ These discrepancies may be due to the relatively low sugar intake and distinct dietary patterns in that cohort. Indeed, nutrient profiles high in energy and sugar have been associated with lower BMD, while healthier patterns are linked to better bone health.^[23]^

Several potential mechanisms may explain the observed associations. First, excessive sugar intake can adversely affect bone metabolism by increasing urinary calcium excretion, impairing the absorption of active vitamin D, and disrupting calcium homeostasis.^[24,25]^ Additionally, high sugar consumption may promote chronic low-grade inflammation through the upregulation of pro-inflammatory cytokines such as IL-6 and TNF-α, which can exacerbate bone resorption and systemic deterioration.^[26,27]^ Second, diets high in sugar are closely linked to insulin resistance, which may inhibit osteoblast function and disturb calcium-phosphorus metabolism, thereby accelerating bone loss (especially in individuals with osteoporosis).^[28]^ Furthermore, dysregulated sugar metabolism can elevate oxidative stress levels, a key factor implicated in osteoporosis and other age-related conditions.^[29]^ Nonetheless, the precise biological mechanisms through which excessive sugar intake harms bone health and increases mortality risk warrant further investigation.

In our subgroup analysis, the positive link between total sugar intake and all-cause mortality was weaker in females with osteopenia or osteoporosis. A Japanese study similarly found this association only in men, not women.^[30]^ Conversely, sugar-sweetened beverage intake was linked to lower bone mineral density and higher fracture risk specifically in women, especially postmenopausal.^[21,31,32]^ Estrogen exerts a protective effect on bone metabolism, and its decline after menopause accelerates bone loss, potentially contributing to the sex-specific differences observed.^[33,34]^

Despite the strengths of this study, several limitations should be acknowledged. First, the retrospective cohort design limits the ability to establish causal relationships and fully eliminate confounding bias. Second, dietary intake was assessed through self-reported 24-hour recalls, which are subject to recall bias and may not capture changes in eating behavior. Third, we did not differentiate between sources or types of sugar intake, which may have influenced the associations observed. Fourth, although multiple covariates were adjusted for, residual confounding from unmeasured factors cannot be ruled out. Additionally, disease histories were based on self-reported diagnoses, potentially introducing misclassification bias. Finally, as the study population was limited to U.S. adults, the findings may not be generalizable to other populations.

## 5. Conclusions

Higher total sugar intake was associated with an increased risk of all-cause mortality among individuals with osteopenia or osteoporosis, but not among those with normal bone mineral density. These findings suggest that individuals with compromised bone health may be more vulnerable to the adverse effects of dietary sugar, underscoring the importance of sugar reduction as a potential modifiable target in strategies aimed at lowering mortality risk in this population.

## Acknowledgments

The author thanks the staff and the participants of the NHANES study for their valuable contributions.

## Author contributions

**Conceptualization:** Min Liang.

**Data curation:** Tingting Chen, Min Liang.

**Formal analysis:** Tingting Chen.

**Investigation:** Tingting Chen, Min Liang.

**Methodology:** Tingting Chen.

**Supervision:** Min Liang.

**Validation:** Tingting Chen, Min Liang.

**Visualization:** Tingting Chen, Min Liang.

**Writing – original draft:** Tingting Chen, Min Liang.

**Writing – review & editing:** Tingting Chen, Min Liang.

## Supplementary Material



## References

[1] Ensrud KE Crandall CJ. Osteoporosis. Ann Intern Med. 2017;167:ITC17–32. 28761958 10.7326/AITC201708010

[2] Wright NC Looker AC Saag KG. The recent prevalence of osteoporosis and low bone mass in the United States based on bone mineral density at the femoral neck or lumbar spine. J Bone Miner Res. 2014;29:2520–6. 24771492 10.1002/jbmr.2269 PMC4757905

[3] Guo D Zhao M Xu W He H Li B Hou T. Dietary interventions for better management of osteoporosis: an overview. Crit Rev Food Sci Nutr. 2023;63:125–44. 34251926 10.1080/10408398.2021.1944975

[4] Huang Y Chen Z Chen B. Dietary sugar consumption and health: umbrella review. BMJ. 2023;381:e071609. 37019448 10.1136/bmj-2022-071609 PMC10074550

[5] Phillips JA. Dietary guidelines for Americans, 2020–2025. Workplace Health Saf. 2021;69:395. 34279148 10.1177/21650799211026980

[6] WHO Guidelines Approved by the Guidelines Review Committee. Guideline: Sugars Intake for Adults and Children. World Health Organization; 2015. 25905159

[7] Looker AC Orwoll ES Johnston CC Jr. Prevalence of low femoral bone density in older U.S. adults from NHANES III. J Bone Miner Res. 1997;12:1761–8. 9383679 10.1359/jbmr.1997.12.11.1761

[8] Kanis JA. Assessment of fracture risk and its application to screening for postmenopausal osteoporosis: synopsis of a WHO report. WHO Study Group. Osteoporos Int. 1994;4:368–81. 7696835 10.1007/BF01622200

[9] Johnell O Kanis JA. An estimate of the worldwide prevalence and disability associated with osteoporotic fractures. Osteoporos Int. 2006;17:1726–33. 16983459 10.1007/s00198-006-0172-4

[10] Zhang J Feng Y Yang X. Dose-response association of dietary inflammatory potential with all-cause and cause-specific mortality. Adv Nutr. 2022;13:1834–45. 35524691 10.1093/advances/nmac049 PMC9526847

[11] Naomi ND Brouwer-Brolsma EM Buso MEC. Association of sweetened beverages consumption with all-cause mortality risk among Dutch adults: the Lifelines Cohort Study (the SWEET project). Eur J Nutr. 2023;62:797–806. 36271197 10.1007/s00394-022-03023-6 PMC9589708

[12] Shi Z Zhu W Lei Z. Intake of added sugar from different sources and risk of all-cause mortality and cardiovascular diseases: the role of body mass index. J Nutr. 2024;154:3457–64. 39307279 10.1016/j.tjnut.2024.09.017

[13] Laguna JC Alegret M Cofán M. Simple sugar intake and cancer incidence, cancer mortality and all-cause mortality: a cohort study from the PREDIMED trial. Clin Nutr. 2021;40:5269–77. 34536637 10.1016/j.clnu.2021.07.031

[14] Farvid MS Barnett JB Spence ND Rosner BA Holmes MD. Types of carbohydrate intake and breast cancer survival. Eur J Nutr. 2021;60:4565–77. 34152461 10.1007/s00394-021-02517-z PMC9938676

[15] Zoltick ES Smith-Warner SA Yuan C. Sugar-sweetened beverage, artificially sweetened beverage and sugar intake and colorectal cancer survival. Br J Cancer. 2021;125:1016–24. 34267328 10.1038/s41416-021-01487-7 PMC8476625

[16] Jessri M Hennessey D Bader Eddeen A. Sodium, added sugar and saturated fat intake in relation to mortality and CVD events in adults: Canadian National Nutrition Survey linked with vital statistics and health administrative databases. Br J Nutr. 2023;129:1740–50. 35392993 10.1017/S000711452200099X PMC10099775

[17] Sanmartin C Decady Y Trudeau R. Linking the Canadian Community Health Survey and the Canadian Mortality Database: an enhanced data source for the study of mortality. Health Rep. 2016;27:10–8. 28002578

[18] Farhat GN Cauley JA. The link between osteoporosis and cardiovascular disease. Clin Cases Miner Bone Metab. 2008;5:19–34. 22460842 PMC2781192

[19] Musso G Paschetta E Gambino R Cassader M Molinaro F. Interactions among bone, liver, and adipose tissue predisposing to diabesity and fatty liver. Trends Mol Med. 2013;19:522–35. 23816817 10.1016/j.molmed.2013.05.006

[20] Fan Z Zhao J Chen J Hu W Ma J Ma X. Causal associations of osteoporosis with stroke: a bidirectional Mendelian randomization study. Osteoporos Int. 2024;35:2127–35. 39180677 10.1007/s00198-024-07235-w

[21] Ahn H Park YK. Sugar-sweetened beverage consumption and bone health: a systematic review and meta-analysis. Nutr J. 2021;20:41. 33952276 10.1186/s12937-021-00698-1 PMC8101184

[22] Liu ZM Tse SLA Chen B. Dietary sugar intake does not pose any risk of bone loss and non-traumatic fracture and is associated with a decrease in all-cause mortality among Chinese elderly: finding from an 11-year longitudinal study of Mr. and Ms. OS Hong Kong. Bone. 2018;116:154–61. 30010084 10.1016/j.bone.2018.07.011

[23] Mazidi M Kengne AP Vatanparast H. Association of dietary patterns of American adults with bone mineral density and fracture. Public Health Nutr. 2018;21:2417–23. 29779504 10.1017/S1368980018000939 PMC10260878

[24] DiNicolantonio JJ Mehta V Zaman SB O’Keefe JH. Not salt but sugar as aetiological in osteoporosis: a review. Mo Med. 2018;115:247–52. 30228731 PMC6140170

[25] Terada M Inaba M Yano Y. Growth-inhibitory effect of a high glucose concentration on osteoblast-like cells. Bone. 1998;22:17–23. 9437509 10.1016/s8756-3282(97)00220-2

[26] Ma X Nan F Liang H. Excessive intake of sugar: an accomplice of inflammation. Front Immunol. 2022;13:988481. 36119103 10.3389/fimmu.2022.988481 PMC9471313

[27] Torres HM Arnold KM Oviedo M Westendorf JJ Weaver SR. Inflammatory processes affecting bone health and repair. Curr Osteoporos Rep. 2023;21:842–53. 37759135 10.1007/s11914-023-00824-4 PMC10842967

[28] Sharma P Sharma RK Gaur K. Understanding the impact of diabetes on bone health: a clinical review. Metabol Open. 2024;24:100330. 39606009 10.1016/j.metop.2024.100330 PMC11600011

[29] Cavati G Pirrotta F Merlotti D. Role of advanced glycation end-products and oxidative stress in type-2-diabetes-induced bone fragility and implications on fracture risk stratification. Antioxidants (Basel). 2023;12:928. 37107303 10.3390/antiox12040928 PMC10135862

[30] Sasakabe T Wakai K Ukawa S. Food group intakes and all-cause mortality among a young older Japanese population of the same age: the New Integrated Suburban Seniority Investigation Project. Nagoya J Med Sci. 2021;83:169–82. 33727748 10.18999/nagjms.83.1.169 PMC7938093

[31] Fung TT Arasaratnam MH Grodstein F. Soda consumption and risk of hip fractures in postmenopausal women in the Nurses’ Health Study. Am J Clin Nutr. 2014;100:953–8. 25099544 10.3945/ajcn.114.083352 PMC4135502

[32] Kremer PA Laughlin GA Shadyab AH. Association between soft drink consumption and osteoporotic fractures among postmenopausal women: the Women’s Health Initiative. Menopause. 2019;26:1234–41. 31613830 10.1097/GME.0000000000001389

[33] Gosset A Pouillès JM Trémollieres F. Menopausal hormone therapy for the management of osteoporosis. Best Pract Res Clin Endocrinol Metab. 2021;35:101551. 34119418 10.1016/j.beem.2021.101551

[34] Alswat KA. Gender disparities in osteoporosis. J Clin Med Res. 2017;9:382–7. 28392857 10.14740/jocmr2970w PMC5380170

