# Supplementary material for: Association between total sugar intake and all-cause mortality according to bone health status: A retrospective cohort study
Source: Medicine (Baltimore). 2026 Jan 23;105(4):e46133. doi: 10.1097/MD.0000000000046133 (PMC12851782; doi:10.1097/MD.0000000000046133)
Supplement: Supplementary file 1 [file medi-105-e46133-s001.docx]

Supplementary Tables 1 Subgroup analysis of mortality among participants with osteopenia or osteoporosis

| All-cause mortality | HR (95% CI),Per 10 units increase | P value | P for interaction |
| --- | --- | --- | --- |
| Gender |  |  | 0.041 |
| Male | 1.040 (1.022, 1.058) | <0.001 |  |
| Female | 1.014 (0.992, 1.036) | 0.219 |  |
| Age |  |  | 0.172 |
| <65 | 1.036 (1.014, 1.058) | 0.001 |  |
| ≥65 | 1.016 (1.000, 1.032) | 0.045 |  |
| BMI |  |  | 0.428 |
| <25 | 1.023 (1.003, 1.043) | 0.026 |  |
| ≥25 | 1.034 (1.017, 1.053) | <0.001 |  |
| Diabetes mellitus |  |  | 0.535 |
| No | 1.025 (1.009, 1.042) | 0.002 |  |
| Yes | 1.046 (1.021, 1.071) | <0.001 |  |
| Hypertension |  |  | 0.910 |
| No | 1.035 (1.011, 1.059) | 0.004 |  |
| Yes | 1.027 (1.010, 1.044) | 0.002 |  |

Model was adjusted for age, gender, race, educational level, marital status, poverty income ratio, smoking statues, drinking status and physical activity, BMI, medical history of hypertension, cardiovascular disease, diabetes mellitus, chronic kidney disease, dyslipidemia, cancer, fracture and use of glucocorticoid, but the model did not adjust for the stratification variables themselves.

Supplementary Tables 2 Subgroup analysis of mortality among participants with normal bone density

| All-cause mortality | HR (95% CI),Per 10 units increase | P value | P for interaction |
| --- | --- | --- | --- |
| Gender |  |  | 0.659 |
| Male | 0.998 (0.981, 1.016) | 0.832 |  |
| Female | 1.011 (0.979, 1.043) | 0.508 |  |
| Age |  |  | 0.927 |
| <65 | 1.002 (0.985, 1.020) | 0.791 |  |
| ≥65 | 0.999 (0.974, 1.025) | 0.955 |  |
| BMI |  |  | 0.332 |
| <25 | 0.997 (0.964, 1.031) | 0.873 |  |
| ≥25 | 1.004 (0.990, 1.018) | 0.609 |  |
| Diabetes mellitus |  |  | 0.677 |
| No | 0.998 (0.981, 1.016) | 0.857 |  |
| Yes | 1.008 (0.980, 1.036) | 0.581 |  |
| Hypertension |  |  | 0.993 |
| No | 1.000 (0.976, 1.025) | 0.983 |  |
| Yes | 1.001 (0.985, 1.018) | 0.879 |  |

Model was adjusted for age, gender, race, educational level, marital status, poverty income ratio, smoking statues, drinking status and physical activity, BMI, medical history of hypertension, cardiovascular disease, diabetes mellitus, chronic kidney disease, dyslipidemia, cancer, fracture and use of glucocorticoid, but the model did not adjust for the stratification variables themselves.
